# Rat intestinal homogenate and pancreatic juice can induce the *Z*-isomerization of all-*E*-lycopene *in vitro*

**DOI:** 10.1038/s41598-020-67093-4

**Published:** 2020-06-19

**Authors:** Jin Huang, Bodi Hui

**Affiliations:** 1 Department of Food Science, Beijing Union University, Beijing, 100023 P.R. China; 2 COFCO Nutrition and Health Research Institute Co.,Ltd., Beijing, 102209 P.R. China

**Keywords:** Mass spectrometry, Bioanalytical chemistry

## Abstract

Lycopene is one of the carotenoids often consumed by humans in their diet. Although lycopene exists mainly in the form of the all *E*-isomer in foods, the considerable quantity of its *Z*-isomers is found in the human plasma and liver. This observation suggested that the lycopene all-*E*-isomer was converted into *Z*-isomers in the human body. In this study, the *Z*-isomerization of the all-*E*-isomer was induced *in vitro* by the pancreatic juice and small intestinal homogenate of male rats under the conditions of 37 °C, pH = 7.5, nitrogen and darkness, as well as shaking. After 2 hours, the proportion of the all *E*-isomer decreased to 25% and *Z*-isomer amounts increased relatively. The converted products were identified as 5, 9, and 11 *Z*-isomers by electronic absorption spectroscopy and mass spectrometry (MS). The observations from this experiment suggested that the *Z*-isomerization site of the lycopene all *E*-isomer was located in the small intestinal wall of the rat.

## Introduction

**Figure 1:**
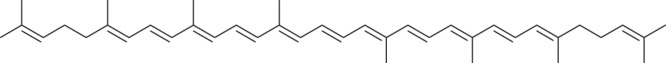
Molecular structure of all-*E* lycopene.

Lycopene is a major carotenoid extensively found in tomato fruits. As shown in Fig. 1, the lycopene molecule consists of a central polyene chain with 11 conjugated double bonds and two identical acyclic (*ψ*) end groups with a non-conjugated double bond in each. Therefore, lycopene occurs as either all-*E*-isomer or various *Z*-isomers and exhibits a strong singlet oxygen quenching ability. In higher plants and microorganisms, the lycopene all *E*-isomer is bio-synthesized and accumulates in large quantities, while 5, 9, 11, and 13 *Z*-isomers are found in small amounts.

The existence of lycopene geometrical isomers has been confirmed for a long time. Zechmeister *et al*. recorded the geometrical isomerization of all *E*-lycopene in 1938^1^. In the 1980s, the C30 stationary phase was employed on High Performance Liquid Chromatography (HPLC), while Emenhiser *et al*. reported a reliable lycopene geometrical isomer separation method on C30-HPLC^2–4^. After separation, the following approaches were employed for lycopene isomer structure elucidation: (1) The comparison of fraction chromatographic behaviors such as retention time and elution sequence with those of reference^3,4]^; (2) Electronic absorption spectroscopy^5,6^; (3) Nuclear Magnetic Resonance (NMR)^7,8^ and (4) Melting point determination^8^. However, *Z*-isomers have not become commercially available yet. Therefore, it is currently difficult to quantify the *Z*-isomer fraction on C30-HPLC. Although the electronic absorption coefficients of the *Z*-isomers are still unknown, in practice, the all *E*-isomer is typically used as a reference sample to quantify the *Z*-isomer fraction on C30-HPLC equipped with a ultraviolet-visible detector.

The intake, absorption, transportation, storage, and metabolism of lycopene in the human body can be summarized as follows: orally administered lycopene is absorbed into the plasma in the small intestine and transported to the target organ while some of them are transported to the liver for storage. A similar process is also found in most mammals such as rats.

Due to its various health functions *in vivo*, lycopene is consumed in large quantities by mammals including humans. Data from the geometrical isomer composition assessment suggested that most lycopene molecules occurred as all *E*-isomers in foods. By contrast, nearly half of lycopene molecules exist as *Z*-isomers in the plasma^9–11^ and other tissues, such as the liver^12,13^ of mammals including humans. This observation is further confirmed in different populations^14,15^, and suggest that a significant *Z*-isomerization of all *E*-isomer takes place during the absorption, transportation, and storage of lycopene molecules.

The *Z*-isomerization site in the human body is always a concern. Existing evidence suggests that the *Z*-isomerization of all *E*-isomers is likely to occur at different sites *in vivo*, such as the small intestine^16,17^, the plasma^18–20^ and the liver^21^. In other words, the *Z*-isomerization of all *E*-isomer might occur before absorption, as well as during transportation and storage in the human body. Moreover, isomerization is reversible^22^.

The factors affecting the lycopene isomer composition of the human plasma and liver have also attracted attention. It was proved that the bioavailability of *Z*-isomers was higher than that of all-*E*-isomers^23–26^. Furthermore, the isomer composition of lycopene from foods could affect that of lycopene from human plasma, liver and other tissues^27–33^.

An attempt was made in this study to prove that the *Z*-isomerization site of all *E*-isomers was located at the small intestinal wall of rats. In the experiment, the *Z*-isomerization of all *E*-lycopene emulsified with Tween 80 in the pancreatic juice, and was then induced by small intestinal homogenate from male rats. Data from this study provided evidence to prove that both the small intestinal intracellular chemical environment and the pancreatic juice were necessary for *Z*-isomerization. Electronic absorption spectroscopy and mass spectrometry identified the induced products as lycopene 5, 9, and 11 *Z*-isomers.

## Materials and methods

### Materials

All *E*-lycopene reference samples were purchased from MERCK (P/N: SMB00706-1MG, 1 mg/Ampere bottle) with a purity of >98% (W/W, C18-HPLC) and the all *E*-isomer content being more than 99.6% (W/W, C30-HPLC). All *E*-lycopene as the reaction substrate was prepared from tomatoes and was provided by Xinjiang Tomato Red Biotechnology Co., Ltd. This had a purity of >96% (W/W, C18-HPLC) and the all *E*-isomer proportion was more than 97% (C30-HPLC). In practice, the latter was dissolved in a small amount of dichloromethane (DCM) and then in a large amount of petroleum ether (PE). The dissolved lycopene was further purified on a neutral alumina column with PE as eluent to remove epoxides. The eluent was finally removed by rotary evaporation at −0.06 MPa, 40 °C, and 30 rpm under strict dark conditions. The residues were collected and stored in nitrogen at −80 °C.

Sodium chloride, Tween 80, DCM, acetone, PE and hexane were all Analytical Reagent (AR)-grade reagents and purchased from MERCK. Acetonitrile (CH_3_CN), methanol (MeOH) and methyl tert-butyl ether (MTBE) used in the HPLC mobile phase were all HPLC-grade reagents and purchased from Dikma Technologies (Richmond Hill, ON, USA). Soybean oil was purchased from a local supermarket. Sodium pentobarbital for injection was purchased from a local pharmacy.

C18-HPLC was used to verify and confirm that the regular feeds were lycopene free.

Experiments were performed using adult male rats (12 weeks old and 300 ± 10 g BW) from the Beijing Vital River Laboratory Animal Technology Co., Ltd. (Animal License: SCXK (Beijing) 2016-0002). All experimental protocols for animals were performed in accordance with the Guidelines for Animal Experiments at Beijing Union University. This was approved and licensed by the Animal Research and Ethics Committee of Laboratory Animals, Health Food Function Verification Center, College of Applied Arts and Sciences and Beijing Union University.

The rats were individually housed in plastic cages in a SPF animal house under the following conditions: a 12 h light/12 h dark cycle (lights on 9:00AM to 9:00PM) with constant temperature (22 ± 1 °C) and relative humidity (48 ± 2%). All rats received sufficient amounts of regular feeds and water for 3 days before the single-dose oral administration of lycopene-soybean solution. C18-HPLC was employed to verify and confirm that the feeds were lycopene free.

## Methods

### Solution preparation

A 250 mL 0.3% (W/V) sodium pentobarbital solution was prepared and filtered with a 0.22 micrometer membrane. A 1000 mL 0.9% (W/W) normal saline was prepared. Under strict dark conditions, 112.5 mg purified lycopene (all-*E*-lycopene content >97%, W/W) was accurately (up to 0.00001 g) weighed in a mortar. Soybean oil was gradually added and ground to prepare 150 mL lycopene-soybean oil solution at the all *E*-lycopene concentration of 0.75 mg/mL.

### Single-dose oral administration of lycopene

Male rats may have individual differences in physiology and metabolism. The chemical compositions of pancreatic juice and small intestinal wall tissues of male rats are affected by the changes of rat physiological conditions. Due to these reasons, before collecting pancreatic juice and small intestinal wall tissues, 1 mL of lycopene soybean oil solution (0.75 mg/mL) was orally administrated with a single-dose in order to decrease a variation in the chemical compositions of pancreatic juice and small intestinal wall tissues between the rats.

Before this experiment, the authors performed a number of pre-experiments in the pharmacokinetics of lycopene. It was observed that the maximum lycopene amount from serum appeared at 2 hours after the lycopene oral administration. This observation suggested that the absorption of male rats were under good conditions at 2 hours. In this experiment, pancreatic juice and small intestinal wall tissues were collected at 2 hours after the oral administration for the induction of lycopene *E*/*Z* conversion *in vitro*.

### Pancreatic juice collection and sterilization

Two hours after the single-dose oral administration of lycopene, 50 rats were selected for pancreatic juice collection. Each rat was intraperitoneally injected with 2.0 mL 0.3% sodium pentobarbital solution. After the rat was anesthetized, the belly was shaved. A 2.0 cm incision was made below the sternal xiphoid process to expose the pancreaticobiliary duct. Under the field of surgical microscopy, the pancreaticobiliary duct was dissected, ligated near the duodenum and the liver, respectively, and intubated. The pancreatic juice was collected after the fluid color changed from yellow to colorless and transparent. Pancreatic juice collection usually takes 2 hours, and more than 1.5 mL pancreatic juice can be collected from each rat. The pH of collected pancreatic juice was 7.5. The collected pancreatic juice from each rat was combined in a volumetric cylinder. 20% (V/V) of the combined pancreatic juice were sterilized under the conditions of 121 °C, 103.4 KPa for 15 minutes.

### Lycopene emulsion preparation

Under strict dark condition, 30 mg of purified lycopene (all *E*-lycopene content >97%, W/W) was accurately (up to 0.00001 g) weighed in a mortar. The mortar was placed in an ice bath, and 4.0 mL of DCM was added and ground for 30 s. 2.0 mL of Tween 80 was then added and ground for 3 minutes. The collected pancreatic juice was gradually added and ground to prepare 60 mL all *E*-lycopene-pancreatic juice emulsion at the all *E*-lycopene concentration of 0.5 mg/mL and pH = 7.5. The sterilized pancreatic juice was gradually added and ground to prepare 60 mL all *E*-lycopene-sterilized pancreatic juice emulsion at the all *E*-lycopene concentration of 0.5 mg/mL and pH = 7.5. Under the same conditions, the pancreatic juice was replaced by the same volume of normal saline to prepare 60 mL all *E*-lycopene-normal saline emulsion at the all *E*-lycopene concentration of 0.5 mg/mL and pH = 7.5.

### Small intestinal homogenate preparation

Two hours after lycopene oral administration, 15 rats were selected for the small intestine collection. After each rat was sacrificed, 4.25 cm duodenum and 92.00 cm jejunum and ileum (96.25 cm in total) were immediately taken from the animal to obtain 5.1000 ± 0.2000 g of the small intestinal segment. The collected intestinal segments were cut axially and drip washed with normal saline to remove feed residues completely from the small intestine wall. During the drip washing process, the dropping pipette was prohibited from touching the inner wall of the small intestinal segment to prevent the villus structure damage. The clean segment of the small intestine was homogenized with a glass homogenizer in an ice bath to produce 60 g small intestinal homogenate. The homogenate was disrupted by the ultrasonic cell disruptor in an ice bath for 3 times. Each disruption was performed at 500 W for 10 min.

### Z-isomerization induction

Five reaction mixtures were prepared as follows: (1) 30 mL normal saline was added to 30 mL lycopene-normal saline emulsion (pH = 7.5); (2) 30 mL lycopene-normal saline emulsion was added to 30 g small intestinal homogenate (pH = 7.4); (3) 30 mL normal saline was added to 30 mL lycopene-pancreatic juice emulsion (pH = 7.4); (4) 30 mL lycopene-pancreatic juice emulsion was added to 30 g small intestinal homogenate (pH = 7.4) and (5) 30 mL lycopene-sterilized pancreatic juice emulsion was added to 30 g small intestinal homogenate (pH = 7.4). All mixtures were vortexed for three times, 15 seconds each. Each mixture was incubated in a 100-mL conical flask at 36.8 ± 0.2 °C with shaking at 15 rpm for 4 hours in the dark and with the presence of nitrogen. The lycopene levels and geometrical isomer compositions were measured hourly.

### Extraction of the reaction products

Five grams of the reaction product was taken from the incubated mixture every hour. The product was mixed thoroughly with 5 mL acetone via the vortex. The vortex was repeated for three times, 15 seconds each, followed by the addition of 10 mL n-hexane and then vortexed three times, 15 seconds each. The mixture was centrifuged at 3000 rpm for 3 minutes. The upper phase was collected while the lower phase was re-extracted twice with 5 mL n-hexane. All upper phases were combined and washed with 20 mL distilled water in a separating funnel and then collected in a 25 mL brown volumetric flask. The volume was scaled with n-hexane. The solution stood at −20 °C overnight, before collecting 4 mL supernatant for lycopene content determination and electronic absorption spectrum acquisition by the ultraviolet-visible spectrophotometer. Following the filtration of 2 mL supernatant through a 0.45 μm filter, the filtrate was collected for analytical HPLC assessment. The control sample was extracted and purified followed the same procedure above.

To prepare the sample for the preparative HPLC separation of reaction products, the same extraction and purification processes were undertaken as above while 30 g reaction product was needed for extraction after the 4-hour incubation. In the final stage, after the extracts stood overnight at −20 °C, the supernatant was filtered to remove lipid precipitates, and was then concentrated to 25 mL using the rotary evaporator at −0.06 MPa, 40 °C and 30 rpm. The concentrate was passed through a 0.45 μm filter and collected for preparative HPLC separation.

### Lycopene amount measurement of the extracts

The lycopene amount in the extracts was measured by the electronic absorption spectrophotometer with the external reference. The ultraviolet-visible spectrophotometer used in this investigation was a Multispec-1501 with a 1*1*4 cm cuvette from SHIMADZU. The 25 mL stock solution of the all-*E*-lycopene reference sample was prepared with petroleum ether at the concentration of 40 μg/mL. Seven working solutions were prepared via double dilution with petroleum ether at concentrations of 0.039 μg/ml, 0.078 μg/ml, 0.156 μg/ml, 0.313 μg/ml, 0.625 μg/ml, 1.250 μg/ml, and 2.500 μg/ml respectively. With petroleum ether as the blank, the absorbance of each working solution was measured at 472 nm on the spectrophotometer. A concentration-absorbance linear correlationship (over 0) was regressed, and the R^2^ value was calculated. The absorbance of each extract was measured at 472 nm. The amount of lycopene from each extract was calculated according to the regression equation.

### Lycopene geometrical isomer composition determination of the extracts

The lycopene geometrical isomer composition from each extract was determined with analytical C30-HPLC. A binary gradient HPLC from Waters Associates was employed in this study and consisted primarily of a Waters 600 E solvent delivery system, a Waters 2996 detector (photo-diode array), an injector with 20 μL and 1 mL sample loops for analytical and preparative HPLC respectively, as well as an Empower pro data processing program. The system reproducibility was carefully verified before use. The HPLC conditions were as follows: Column: YMC^TM^ Carotenoid S-5 (4.6 × 250 mm, 5 μm); Mobile phase A: CH_3_CN-MeOH (75:25, V/V); Mobile phase B: MTBE; Linear gradient: Mobile phase B increased from 0 to 55% (V/V) in 8 minutes, and was maintained at 55% (V/V) from 8 to 30 minutes; Flow rate: 1.0 mL/min; Injection volume: 20 μL; photo-diode array signal acquisition range: 300-550 nm; chromatogram monitoring wavelength: 470 nm; Column temperature: 25 °C. The peak area of each fraction was integrated,and the relative amount of each fraction was calculated according to Formula (1)

1 $$R{A}_{i}=\frac{{P}_{i}}{{\sum }_{n}^{1}{P}_{i}}$$

Where

RA_i_ = Relative amount of fraction i

P_i_ = Peak area of fraction i

n = Number of the fraction

### Lycopene geometrical isomer collection from the extract

The lycopene geometrical isomer fraction was collected from the extract using preparative C30-HPLC. The HPLC conditions were as similar as those employed for the Lycopene geometrical isomer composition determination of the extracts: Column: YMC^TM^ Carotenoid S-5 (20 mm × 25 cm, 5 Carotenoid S-5; Linear gradient: Mobile phase B increased from 0 to 55% (V/V) in 8 min and was maintained at 55% (V/V) from 8 to 45 minutes; Flow rate: 7.0 mL/min; Injection volume: 1.0 mL. The injection was repeated for three times, and each fraction was collected and combined. Solvents were removed from the combined fractions under a stream of nitrogen in dark.

### Mass spectrometry (MS)

Electron impact ionization mass spectrometry was employed in this study for lycopene geometrical isomer structure elucidation. For this purpose, the preparative C30-HPLC was used to prepare the isomer fractions sufficiently for electron impact ionization mass spectrometry. The separated isomer fractions were collected from preparative C30-HPLC and dried under a stream of nitrogen. The dried fraction was re-dissolved in DCM for electron impact ionization mass spectrometry. The sample, the isomer-DCM solution was introduced with a direct insertion probe. The mass spectrometer used in this experiment was Thermo Finnigan’s TRACE MS system. The source temperature was 240 °C, the emission current was 200 mA, the electron energy was 70 eV, the scanning range was 50-700 Da (*m/z*), and the scanning time was 4 seconds.

## Results and discussion

### Variation in lycopene amount during the induction

**Figure 2:**
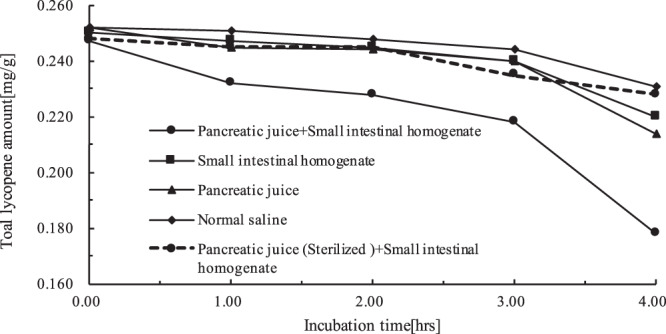
Variation in lycopene amount during the induction.

As shown in Fig. 2 and Table 1, the lycopene level in the reaction products incubated with the pancreatic juice, small intestinal homogenate, and normal saline displayed a slight decrease during the first 3 hours. A significant reduction of lycopene levels was seen in three incubated samples after 3 hours. In comparison, the lycopene level in the reaction product incubated with the pancreatic juice and small intestinal homogenate exhibited an obvious decreased from 1 to 3 hours, and there was a significant reduction after 3 hours as shown in Table 1. If the *Z*-isomers were considered as intermediates in the degradation process of the all-*E*-lycopene molecules^34^, the *Z*-isomerization of all *E*-lycopene should be accompanied by a decrease in the lycopene amount. With the sterilized pancreatic juice and small intestinal homogenate, a slight decrease in lycopene amount was seen during the first 3 hours. This observation suggested that the sterilization inactivated substances from the pancreatic juice. It was observed that only lycopene level in incubate with the pancreatic juice and small intestinal homogenate was reduced remarkably. It was considered that the decrease in lycopene level was likely to be due to the *Z*-isomerization of all-*E*-lycopene. However, this observation was necessary to be confirmed in human and animal models^35^.

**Table 1:** Variation in lycopene amount during the induction (data).

Incubation medium	Lycopene amount [mg/g]				
	Incubation time[hrs]				
	0.00	1.00	2.00	3.00	4.00
+Normal saline	0.252	0.252	0.248	0.244	0.231
+Small intestinal homogenate	0.250	0.247	0.245	0.241	0.223
+Pancreatic juice	0.252	0.245	0.244	0.240	0.214
+Pancreatic juice+ Small intestinal homogenate	0.247	0.232	0.228	0.218	0.178
+Pancreatic juice (Sterilized)+ Small intestinal homogenate	0.248	0.245	0.245	0.235	0.228

### Separation of lycopene geometrical isomers on analytical C30-HPLC

**Figure 3:**
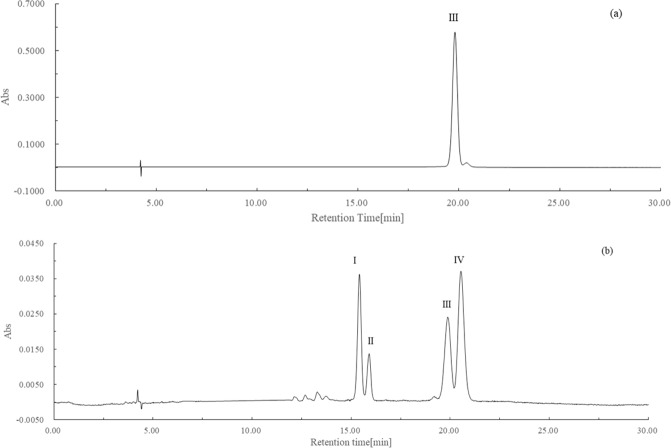
Analytical C30-HPLC profiles of the reaction products induced by pancreatic juice and small intestinal homogenate at 0 h (**a**) and 2 h (**b**) hours.

After the induction, the reaction products contained several lycopene isomers including all *E*-isomers and *Z*-isomers. Those isomers could be readily separated on analytical C30-HPLC under the same conditions to determine the lycopene geometrical isomer composition of the extracts. Figure 3(a,b) illustrate the HPLC profiles of the reaction product extracts at 0 and 2 hours.

### Electronic absorption spectral characteristics of each isomer fraction

**Figure 4:**
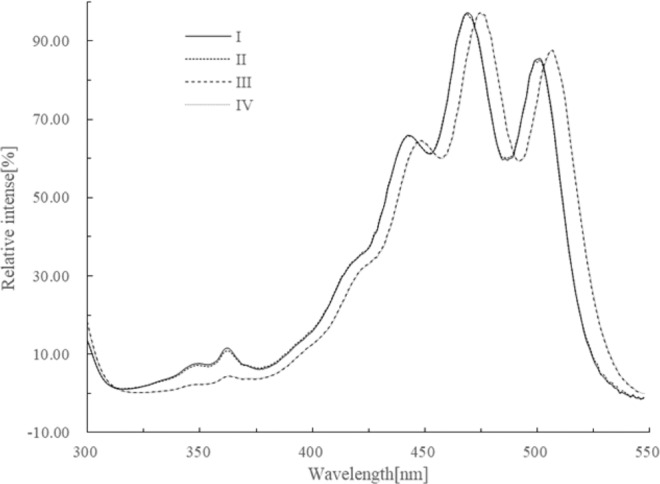
Electronic absorption spectra of fractions I, II, III, and VI. Solvent: HPLC mobile phase.

The electronic absorption spectra of geometrical isomer fractions I, II, III, and IV were acquired from the online photo-diode array detector equipped with HPLC as shown in Fig. 4. Figure 4 indicates that the maximum absorption wavelengths (*max*) of fractions I and II exhibited a slight “hypochromic shift”, while the “*Cis*-peak” absorption intensities of fractions I and II were enhanced compared to those of fraction III. The electronic absorption spectral characteristics of each isomer fraction are summarized in Table 2. Comparing the retention time and electronic absorption spectral characteristics of the product extracts with those of the reference sample and reported data^36^, fraction III was identified as all-*E*-lycopene and fractions I, II and IV were identified as 11 *Z*-, 9 *Z*- and 5 *Z*-isomers of lycopene respectively.

**Table 2:** Electronic absorption spectral characteristics of fractions I, II, III and IV^a^.

Fraction No.	*λ*_*max*_[nm]		Relative *cis*-peak intensity[%]^b^	III/II value[%]^c^
	Major absorption band	*Cis*-peak		
I	468	362	11.81	69.04
II	468	362	11.19	68.04
III	475	363	4.55	74.26
IV	475	363	4.62	75.03

Notes: ^a^Solvents: HPLC mobile phase.

^b^The *cis*-peak and major absorption band intensities at *max* are measured respectively, from the absorbance at 550 nm as the base-line or zero value. The ratio of two measured values is expressed as the relative *cis*-peak intensity [%].

^c^The baseline or zero value is the minimum between the right and middle peaks, while the right and middle peak heights are designated as III and II respectively.

### Separation of lycopene geometrical isomers with preparative C30-HPLC

**Figure 5:**
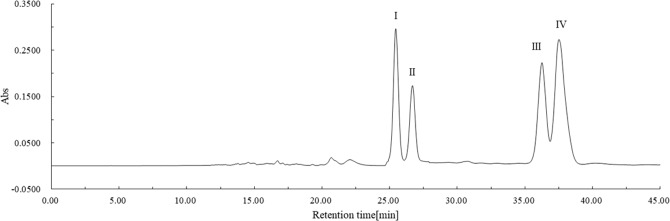
Preparative C30-HPLC profiles of reaction product induced by pancreatic juice and small intestinal homogenate at 4 hours.

The geometrical isomers of lycopene from the reaction product extract at 4 hours were separated thoroughly on preparative C30-HPLC as shown in Fig. 5. A sufficient amount of purified isomer fractions was then collected for electron impact ionization mass spectrometry analysis.

### MS of geometrical isomer fractions I, II, III, and IV

**Figure 6:**
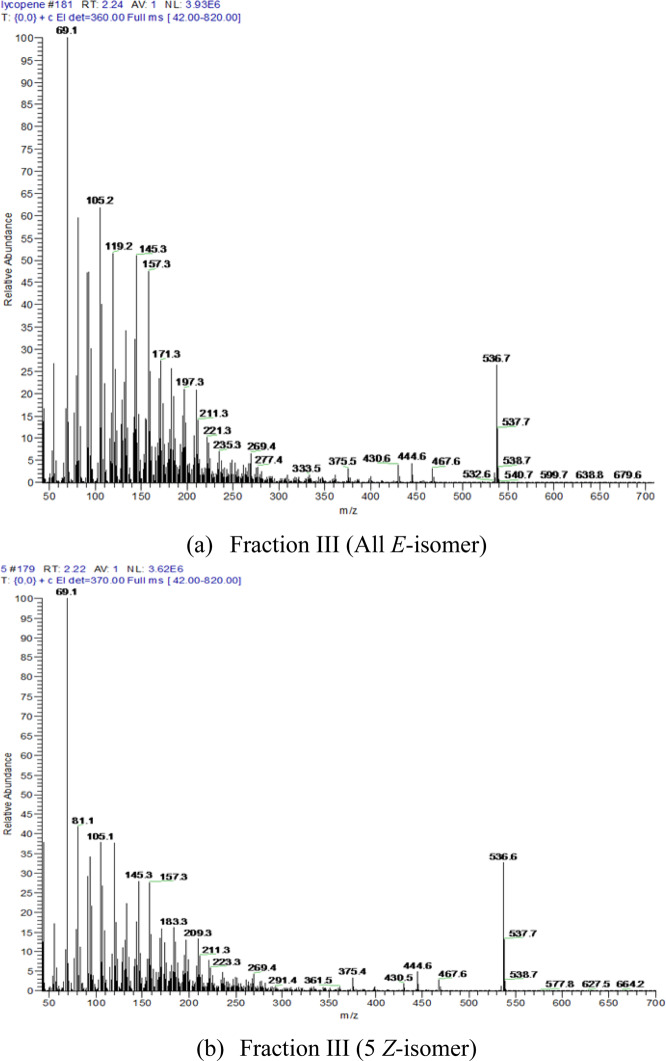
Mass spectra (**a**) and (**b**) of fractions III and IV.

It was observed that all fractions, namely I, II, III and IV had fragments of 55.1 (*m/z*), 69.1 (*m/z*), 105.2 (*m/z*), 269.4 (*m/z*), and 536.7 (*m/z*), while fragment 536.7 was identified as the molecular ion peak [M•]^+^. Mass spectra of fractions III and IV are given in Fig. 6a,b. Fragments 55.1 (*m/z*) and 105.1 (*m/z*) were considered as the products of “Mackintosh rearrangement.” Fragment 69.1 (*m/z*) (100%) was the isoprene [C_5_H_9_•]^+^ fragment. The presence of fragments 69.1 (*m/z*) and 105.1 (*m/z*) suggested that each fraction contained isopentenyl fragments, while the presence of fragment 269.3 (*m/z*) suggested that each fraction was symmetrical. The degree of unsaturation was 13 of the isomers, which meant there were 12 double bonds located in the molecules. Therefore, fractions I, II, III, and IV exhibited the identical elemental composition of C_40_H_56_ and had a molecular mass of 536.7 Da. Those observations suggested that those fractions were the geometrical isomers of each other.

### Variation in lycopene isomer composition during incubation

**Figure 7:**
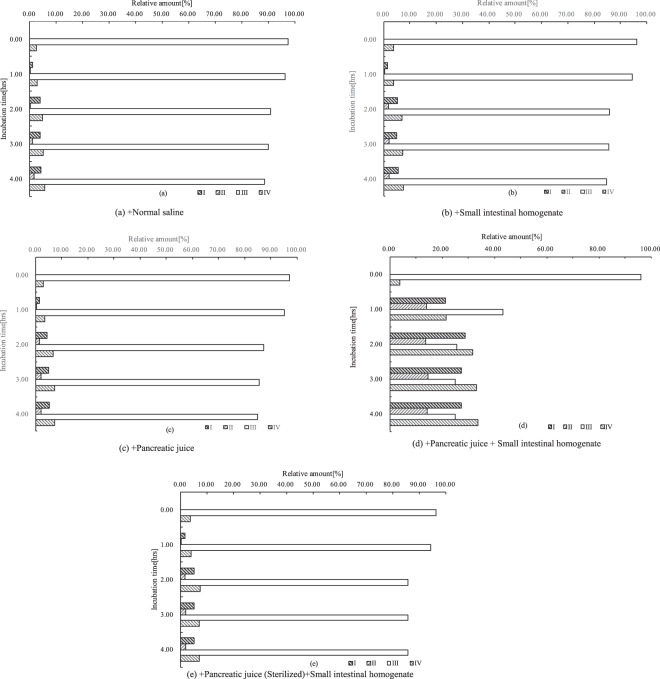
Variation in lycopene geometrical isomer composition during the incubation. The relative amount of each fraction: As calculated according to Formula (1).

According to Formula (1) the relative amount of each isomer fraction was counted from its peak area. The isomer composition of the samples extracted from normal saline incubated (a), small intestinal homogenate induced (b), pancreatic juice induced (c), pancreatic juice and small intestinal homogenate induced (d), and sterilized pancreatic juice and small intestinal homogenate induced (e) reaction products, was further calculated every hour, as shown in Fig. 7 and Table 3(a–e).

**Table 3:** Variation in lycopene geometrical isomer composition during the incubation (data).

Incubation medium	Fraction No.	Relative amount [%]^1^				
(a) +Normal saline		**Incubation time[hrs]**				
		0.00	1.00	2.00	3.00	4.00
	I	0.00	1.01	4.01	3.88	4.30
	II	0.00	0.01	0.35	1.12	1.56
	III	97.34	96.14	90.89	90.02	88.46
	IV	2.66	2.84	4.75	4.98	5.68
(b) +Small intestinal homogenate		**Incubation time[hrs]**				
		0.00	1.00	2.00	3.00	4.00
	I	0.00	1.61	5.19	5.04	5.53
	II	0.00	0.04	1.70	2.14	2.23
	III	96.18	94.56	85.97	85.47	84.68
	IV	3.82	3.79	7.14	7.35	7.56
(c) +Pancreatic juice		**Incubation time[hrs]**				
		0.00	1.00	2.00	3.00	4.00
	I	0.00	1.41	4.34	5.12	5.40
	II	0.00	0.02	1.67	2.08	2.12
	III	97.01	95.12	87.34	85.55	85.03
	IV	2.90	3.45	6.65	7.25	7.45
(d) +Pancreatic juice+ Small intestinal homogenate		**Incubation time[hrs]**				
		0.00	1.00	2.00	3.00	4.00
	I	0.00	21.23	28.84	27.31	27.33
	II	0.00	13.99	13.81	14.56	14.23
	III	96.07	43.11	25.54	24.97	24.88
	IV	3.93	21.67	31.81	33.16	33.56
(e) +Pancreatic juice (Sterilized)+ Small intestinal homogenate		**Incubation time[hrs]**				
		0.00	1.00	2.00	3.00	4.00
	I	0.00	1.69	5.12	5.09	5.21
	II	0.00	0.09	1.72	2.07	2.03
	III	96.34	94.30	85.79	85.68	85.70
	IV	3.66	3.92	7.37	7.16	7.06

Note: ^1^The relative amount of each fraction: as calculated according to Formula (1).

Compared to the product incubated in normal saline as shown in Table 3(a), as seen from Table 3(b,c), the lycopene geometrical isomer compositions of both the small intestinal homogenate and pancreatic juice induced products changed slightly in the first two hours. The proportion of fraction III (all *E*-isomer) decreased slightly while the other fractions (*Z*-isomers) increased relatively. After two hours, the decreased proportion of fraction III was due to a thermal mechanism^33^. These observations suggested that either the small intestinal homogenate or the pancreatic juice was not able to induce a significant geometrical isomerization of all *E*-lycopene. In contrast to observations from Table 3(b,c), it was seen from Table 3(d) that the isomer composition of the reaction product induced by the pancreatic juice and small intestinal homogenate varied significantly in the first and second hours. The proportion of fraction III decreased considerably while the proportion of other fractions increased substantially. Data from this investigation suggested that the *Z*-isomerization of all *E*-lycopene was induced efficiently by the co-action between the pancreatic juice and the small intestine homogenate. When the sterilized pancreatic juice was employed, the isomer composition of the reaction mixture changed slightly during the incubation, as shown in Table 3(e).

Most lycopene from a natural diet exists in all *E*-isomer form while the *Z*-lycopene from human plasma is always significantly higher than that from natural foods^9,10,12^. The major *Z*-isomers from most human plasma are 5*Z*-, 13*Z*- and 9*Z*-isomers in descending order of abundance^37,38^. This indicates that the existence of *Z*-isomers from human plasma is caused by *E*/*Z* conversion but not by selective absorption due to no 13*Z-* and 9*Z*-isomers in our natural diet.

It was reported that the *Z*-isomers of lycopene would be more likely to be incorporated into a bile acid micelle with the decreased tendency of *Z*-isomers to form aggregates, resulting in a higher bioavailability of *Z*-isomers than that of all *E*-isomer^10,11^. Acidic conditions and a fatty environment also affected the absorption and *Z*-isomerization efficiency of lycopene^20,22^. Additionally, the geometrical isomer composition of lycopene in processed foods had certain influence on that in human plasma^27–33^. In plasma, lycopene finally exists as a mixture of geometrical isomers in a certain proportion with a stable thermodynamic equilibrium between isomers^9,15^. Data from Table 3(b,c) suggested that emulsified lycopene all *E*-isomer was not able to be converted into *Z*-isomers when treated with rat pancreatic juice and small intestine homogenate alone. Data shown in Table 3(d) suggested that emulsified all *E*-lycopene could be converted into *Z*-isomers when both the pancreatic juice and small intestine homogenate was applied. Data illustrated in Table 3(e) suggested that higher sterilization would cause an inactivation of pancreatic juice. Data presented as above suggested that the *E/Z*-conversion of *E*-lycopene was likely to take place *in vivo* by the induction of substances from both pancreatic juice and the small intestine wall tissues. The inducers from the pancreatic juice and small intestine homogenate would attract further studies.

Data from the evaluation of various *in vitro* models suggested that *E*-lycopene isomerization did not occur in the stomach, small intestine or in the process of transferring to micelles^18,21,26^. When incorporated into the different clones of Caco-2 cells, lycopene all *E*-isomer was converted into different *Z*-isomers^17^. The proportion of individual *Z*-isomer from Caco-2 cell and human plasma was quite consistent and decreased in the order of 5*Z*- > 13*Z*- > 9*Z*-isomers. The observations from this investigation support a hypothesis that the *E/Z* conversion of lycopene *in vivo* is performed in the wall tissues of the small intestine. This hypothesis was also supported by observations from studies with Caco-2 cells.

## Conclusion

Data from this study suggested that the *Z*-isomerization of all *E*-lycopene emulsified with Tween 80 in rat pancreatic juice was able to be achieved with small intestinal homogenate. This observation provided an evidence to prove that both rat pancreatic juice and small intestinal homogenate offered the chemical conditions necessary for the conversion of lycopene all *E*-isomer into *Z*-isomers. It was therefore considered that this conversion took placed in the small intestinal wall of rat *in vivo*. In other words, all *E*-lycopene were able to be converted into the *Z*-isomers of lycopene when it passed through the small intestinal wall of rats during absorption.

## References

[1] Zechmeister L, Tuzson P (1938). Spontaneous Isomerization of Lycopene. Nature.

[2] Stahl W, Sundquist A, Hanusch M, Schwarz W, Sies H (1993). Separation of *beta*-carotene and lycopene geometrical isomers in biological samples. Clinical Chemistry.

[3] Emenhiser C, Sander L, Schwartz S (1995). Capability of a polymeric C30 stationary phase to resolve *cis*-trans carotenoid isomers in reversed-phase liquid chromatography. Journal of Chromatography A.

[4] Emenhiser C, Simunovic N, Sander L, Schwartz S (1996). Separation of Geometrical Carotenoid Isomers in Biological Extracts Using a Polymeric C30Column in Reversed-Phase Liquid Chromatography. Journal of Agricultural and Food Chemistry.

[5] Fröhlich, Conrad, Schmid, Breithaupt and Böhm. Isolation and Structural Elucidation of Different Geometrical Isomers of Lycopene. International Journal for Vitamin and Nutrition Research, **77**(6), pp.369–375 (2007). 10.1024/0300-9831.77.6.369 18622946

[6] Murillo E (2018). Far UV peaks contribute for identification of carotenoids *E/Z* isomers. Journal of Food Composition and Analysis.

[7] Hengartner U, Bernhard K, Meyer K, Englert G, Glinz E (1992). Synthesis, Isolation, and NMR-Spectroscopic Characterization of Fourteen (*Z*)-Isomers of Lycopene and of Some Acetylenic Didehydro- and Tetradehydrolycopenes. Helvetica Chimica Acta.

[8] Takehara M (2013). Characterization and Thermal Isomerization of (all-E)-Lycopene. Journal of Agricultural and Food Chemistry.

[9] Krinsky N, Russett M, Handelman G, Snodderly D (1990). Structural and Geometrical Isomers of Carotenoids in Human Plasma. The. Journal of Nutrition.

[10] Stahl W, Schwarz W, Sundquist A, Sies H (1992). *cis*-trans isomers of lycopene and β-carotene in human serum and tissues. Archives of Biochemistry and Biophysics.

[11] Ross A (2011). Lycopene bioavailability and metabolism in humans: an accelerator mass spectrometry study. The American Journal of Clinical Nutrition.

[12] Peters U (2007). Serum Lycopene, Other Carotenoids, and Prostate Cancer Risk: a Nested Case-Control Study in the Prostate, Lung, Colorectal, and Ovarian Cancer Screening Trial. Cancer Epidemiology Biomarkers & Prevention.

[13] Boileau T, Clinton S, Erdman J (2000). Tissue Lycopene Concentrations and Isomer Patterns Are Affected by Androgen Status and Dietary Lycopene Concentration in Male F344 Rats. The. Journal of Nutrition.

[14] Boileau, T. *et al*. Lycopene isomers and carotenoid profiles in african american (*aa*) and caucasian (*c*) men. *FASEB J* **12**, A856–A856 (1998).

[15] Wu K (2003). Variations in Plasma Lycopene and Specific Isomers over Time in a Cohort of U.S. Men. The Journal Of Nutrition.

[16] Re R, Fraser P, Long M, Bramley P, Rice-Evans C (2001). Isomerization of Lycopene in the Gastric Milieu. Biochemical And Biophysical Research Communications.

[17] Richelle M (2010). Lycopene isomerisation takes place within enterocytes during absorption in human subjects. British Journal Of Nutrition.

[18] Holloway D, Yang M, Paganga G, Rice-Evans C, Bramley P (2000). Isomerization of dietary lycopene during assimilation and transport in plasma. Free Radical Research.

[19] López-Ramírez M, Sanchez-Cortes S, Pérez-Méndez M, Blanch G (2010). Trans-cis isomerisation of the carotenoid lycopene upon complexation with cholesteric polyester carriers investigated by Raman spectroscopy and density functional theory. Journal Of Raman Spectroscopy.

[20] Colle I, Lemmens L, Van Buggenhout S, Van Loey A, Hendrickx M (2011). Modeling Lycopene Degradation and Isomerization in the Presence of Lipids. Food And Bioprocess. Technology.

[21] Teodoro A, Perrone D, Martucci R, Borojevic R (2009). Lycopene isomerisation and storage in an *in vitro* model of murine hepatic stellate cells. European Journal Of Nutrition.

[22] Moraru C, Lee T (2005). Kinetic Studies of Lycopene Isomerization in a Tributyrin Model System at Gastric pH. Journal Of Agricultural And Food Chemistry.

[23] Boileau T, Boileau A, Erdman J (2002). Bioavailability of all-trans and cis–Isomers of Lycopene. Experimental Biology And Medicine.

[24] Stahl W, Sies H (1992). Uptake of Lycopene and Its Geometrical Isomers Is Greater from Heat-Processed than from Unprocessed Tomato Juice in Humans. The Journal Of Nutrition.

[25] Unlu N (2007). Lycopene from heat-induced *cis*-isomer-rich tomato sauce is more bioavailable than from all-trans-rich tomato sauce in human subjects. British Journal Of Nutrition.

[26] Failla M, Chitchumroonchokchai C, Ishida B (2008). *In Vitro* Micellarization and Intestinal Cell Uptake of cis Isomers of Lycopene Exceed Those of All-trans Lycopene. The Journal Of Nutrition.

[27] Schierle J (1997). Content and isomeric ratio of lycopene in food and human blood plasma. Food Chemistry.

[28] Boileau T (2001). Testosterone and Food Restriction Modulate Hepatic Lycopene Isomer Concentrations in Male F344 Rats. The. Journal of Nutrition.

[29] Alien C, Smith A, Clinton S, Schwartz S (2002). Tomato Consumption Increases Lycopene Isomer Concentrations in Breast Milk and Plasma of Lactating Women. Journal of the American Dietetic Association.

[30] Erdman J (2005). How Do Nutritional and Hormonal Status Modify the Bioavailability, Uptake, and Distribution of Different Isomers of Lycopene?. The Journal of Nutrition.

[31] Fröhlich K, Kaufmann K, Bitsch R, Böhm V (2006). Effects of ingestion of tomatoes, tomato juice and tomato purée on contents of lycopene isomers, tocopherols and ascorbic acid in human plasma as well as on lycopene isomer pattern. British Journal of Nutrition.

[32] Richelle M (2011). The proportion of lycopene isomers in human plasma is modulated by lycopene isomer profile in the meal but not by lycopene preparation. British Journal of Nutrition.

[33] Allen C (2003). Changes in Plasma and Oral Mucosal Lycopene Isomer Concentrations in Healthy Adults Consuming Standard Servings of Processed Tomato Products. Nutrition and Cancer.

[34] Graham D, Carail M, Caris-Veyrat C, Lowe G (2012). *13Z*)- and (*9Z*)-lycopene isomers are major intermediates in the oxidative degradation of lycopene by cigarette smoke and Sin-1. Free Radical Research.

[35] Moran N (2015). Compartmental and noncompartmental modeling of *13C*-lycopene absorption, isomerization, and distribution kinetics in healthy adults. The American Journal of Clinical Nutrition.

[36] Šesták, Z. Britton, G., Liaaen-Jensen, S., Pfander, H. (ed.): Carotenoids. Handbook. Photosynthetica, 42(2), p.186 (2004).

[37] Clinton S (2009). Lycopene: Chemistry, Biology, and Implications for Human Health and Disease. Nutrition Reviews.

[38] Khachik F (2002). Chemistry, Distribution, and Metabolism of Tomato Carotenoids and Their Impact on Human Health. Experimental Biology and Medicine.

